# Investigating the Glycating Effects of Glucose, Glyoxal and Methylglyoxal on Human Sperm

**DOI:** 10.1038/s41598-018-27108-7

**Published:** 2018-06-13

**Authors:** Clare Nevin, Lauren McNeil, Nessar Ahmed, Chris Murgatroyd, Daniel Brison, Michael Carroll

**Affiliations:** 1 School of Healthcare Science, Faculty of Science and Engineering, Manchester Metropolitan University, John Dalton Building, Chester Street, Manchester, M1 5GD UK; 2 Department of Reproductive Medicine, Old St Mary’s Hospital, Manchester University NHS Foundation Trust, Manchester Academic Health Sciences Centre, Oxford Road, Manchester, M13 9PT UK

## Abstract

Glycation is the non-enzymatic reaction between reducing sugars, such as glucose, and proteins, lipids or nucleic acids, producing Advanced Glycation End (AGE) products. AGEs, produced during natural senescence as well as through lifestyle factors such as diet and smoking, are key pathogenic compounds in the initiation and progression of diabetes. Importantly, many of these factors and conditions also have influence on male fertility, affecting sperm count and semen quality, contributing to the decreasing trend in male fertility. This study investigated the impact of AGEs on sperm damage. *In vitro* sperm glycation assays were used to determine the levels and localization of the potent AGE compound, carboxymethyl-lysine (CML) in response to treatment with the glycating compounds glucose, glyoxal and methylglyoxal. Sperm function assays were then used to assess the effects of glycation on motility and hyaluronan binding, and levels of oxidative DNA damage were analyzed through measurement of the marker, 8-oxoguanine. Results showed that glyoxal, but not glucose or methylglyoxal, induced significant increases in CML levels on sperm and this correlated with an increase in 8-oxoguanine. Immunocytochemistry revealed that AGEs were located on all parts of the sperm cell and most prominently on the head region. Sperm motility and hyaluronidase activity were not adversely affected by glycation. Together, the observed detrimental effects of the increased levels of AGE on DNA integrity, without an effect on motility and hyaluronidase activity, suggest that sperm may retain some fertilizing capacity under these adverse conditions.

## Introduction

Many studies over the past few decades have indicated that semen quality has declined^1^, possibly owing to modern lifestyle factors, such as diet, smoking tobacco and drinking alcohol, and the rise in health conditions such as obesity and diabetes^2^. A recent study demonstrated that lifestyle factors, diet and age were positively associated with increased levels of Advanced Glycation End (AGE) Products, which are detrimental to health and reproduction^3^.

**Figure 1:**
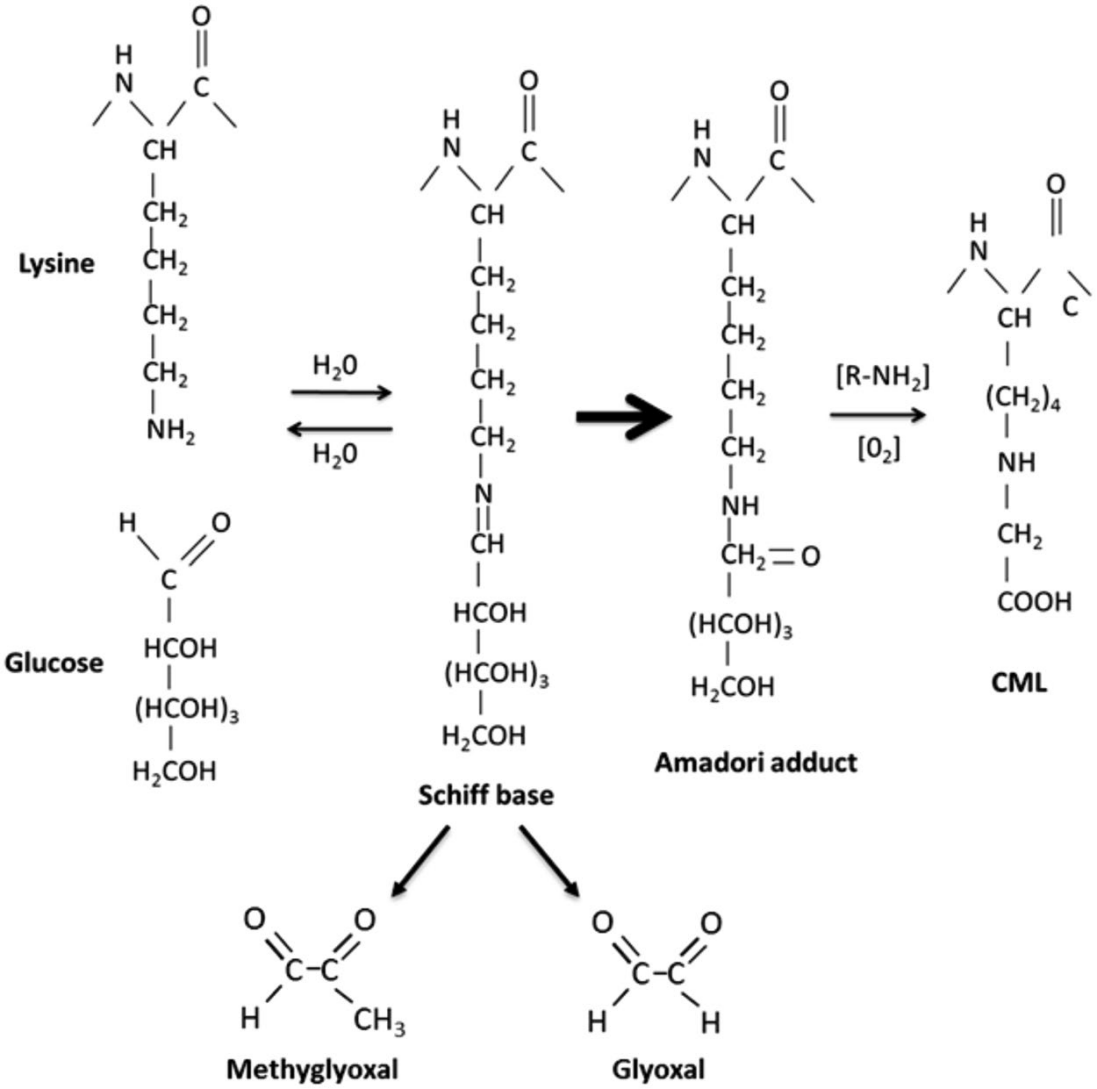
Glycation through the Maillard reaction. AGEs are formed through the Maillard reaction in which the carbonyl group of a reducing sugar, such as glucose, reacts with the amine group of a protein side chain residue, such as lysine. Initially a reversible Schiff base adduct is formed, which then undergoes a series of rearrangements to form an Amadori product. Further glycation and oxidation reactions lead to the formation of a primary AGE Nε-(carboxymethyl)lysine (CML). Fragmentation of the Schiff base generates the AGE intermediates methylglyoxal (MG) and glyoxal (GO). These reactive carbonyl compounds also react with biomolecules to produce AGEs.

AGEs are a heterogeneous group of compounds that form from the non-enzymatic reaction of a carbonyl group of a reducing sugar with the free amino-group of proteins^4^. This process, known as the Maillard reaction^5^, can occur endogenously, where biomolecules such as proteins, lipids and nucleic acids become covalently modified during the process of AGE formation^6^. AGE-modified proteins cause damage to cells and tissues by crosslinking other proteins, such as extracellular matrix proteins, and activating cellular inflammatory response pathways through interaction with the receptor for AGE, RAGE. The level of AGEs is further increased by the action of highly reactive AGE intermediates, including methylglyoxal (MG) and glyoxal (GO), which are formed when AGEs degrade and through Schiff base fragmentation, as summarised in Fig. 1. These intermediates then proceed to form additional AGEs at a faster rate than reducing sugars^7^.

AGE formation is accelerated under conditions including hyperglycaemia, insulin resistance, dyslipidemia and oxidative stress^8–10^ leading to the presence of higher levels in chronic age-related diseases, including cardiovascular disease, diabetic nephropathy, nerve damage, retinopathy and atherosclerosis^11–15^. Increased levels of AGEs within tissues are also associated with consumption of AGE-rich foods, particularly animal-based products high in fat and protein^16,17^, and other life-style factors such as smoking tobacco^18^.

Interestingly, many of the above conditions including diabetes and obesity, and lifestyle exposures such as smoking have also been linked to declines in semen quality and fertility^1,18–21^. Furthermore, the damaging impact of glycation on testicular function has been reported in numerous studies of animal models showing links with Leydig cell function and erectile dysfunction^22–24^. AGEs have been located in the human male reproductive tract, on sperm cells and in soluble form in the seminal plasma^25,26^ suggesting that they may form modifications on functionally important sperm proteins or induce DNA adducts.

Glycation and the formation of AGE can generate reactive oxygen species (ROS). This can happen through AGE-RAGE activation of the NFkB (nuclear factor kappa-light-chain-enhancer of activated B cells) inflammatory pathway leading to subsequent NADPH and ROS production^27^. The presence of AGE therefore has the potential to cause particular damage to sperm due to their high vulnerability to oxidative stress. This is a result of the high level of polyunsaturated fatty acids in the sperm membrane, which undergo lipid peroxidation when levels of ROS exceed the antioxidant capacity of the seminal plasma, leading to cell membrane damage and impaired motility and morphology^28,29^. Furthermore, elevated ROS levels cause sperm DNA fragmentation, a marker of infertility, which is not detected through conventional semen analysis^30^.

AGE and RAGE have previously been detected on sperm and in the male reproductive tract, suggesting that this signalling pathway may have a role in sperm damage. Other studies have demonstrated a role for AGEs in diabetes-related sperm dysfunction^31^. For example, within the seminal plasma of diabetics compared to non-diabetics higher levels of total AGEs were found together with increased lipid peroxidation (malondialdehyde) and reduced total antioxidant capacity^32^. Carboxymethyl-lysine (CML), a common AGE, has been located throughout the seminiferous epithelium and on sperm cells of healthy and diabetic men, with the acrosomal cap being more prominently stained for CML in diabetics^25^. RAGE is also found throughout the testis and the epididymis, as well as on the head region of sperm cells at greater levels in diabetics than non-diabetics^30^ and this directly correlated with sperm DNA fragmentation^31^. The occurrence of AGE and RAGE in healthy males shows that these compounds have some role in sperm function, and their elevated levels in diabetics suggest that they could play a pathological role in these men’s infertility.

The role of AGE in sperm function has not been explored in depth. This study is the first to investigate the *in vitro* effects of glycation on human sperm function, through the assessment of several sperm parameters, including DNA integrity. An *in vitro* assay system was used to delineate a mechanistic link between AGE formation and sperm function and DNA damage. The data presented may pave the way for further *in vivo* studies concerning glycation-induced sperm damage and male infertility.

## Results

### Long-term ***in vitro*** glycation

#### Vitality and motility of sperm after incubation with glycating agents

**Figure 2:**
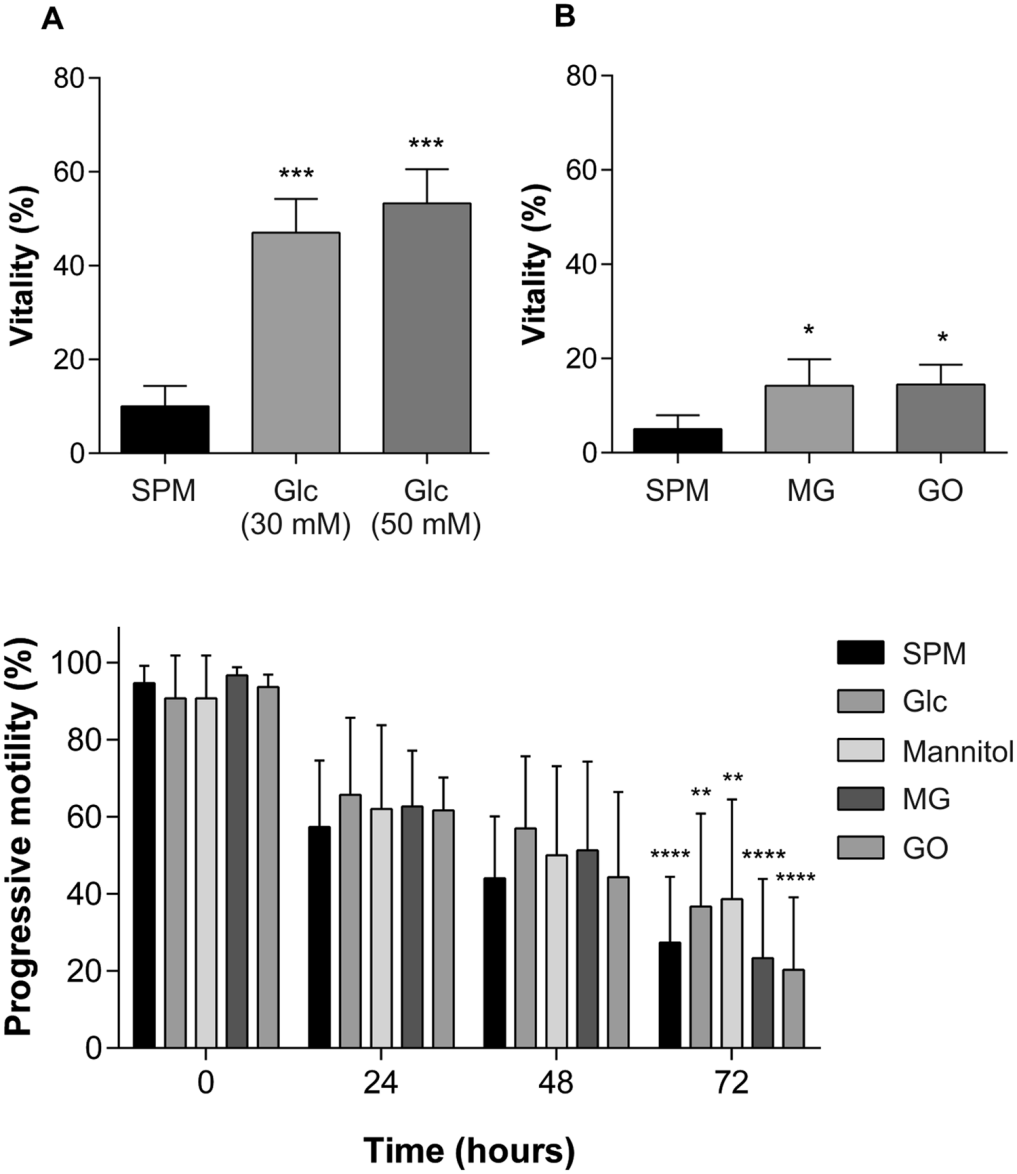
Effect of glycating compounds on sperm vitality and motility. (**A**) The percentage of live sperm following incubation with glucose (Glc) (n = 3) (30 mM and 50 mM), (**B**) methylglyoxal, MG (n = 3) (50 µM) and glyoxal, GO (n = 3) (50 µM) for 6 days, measured using the eosin-nigrosin stain. Sperm vitality decreased in all treatment groups at the end of the incubation period, but the presence of glucose maintained vitality at a significantly higher level than Sperm Preparation Media, SPM (***p < 0.001). The presence of MG and GO also showed significantly higher vitality than SPM (*p < 0.05). (**C**) Sperm progressive motility had significantly decreased after 72 hours for all treatment groups (n = 3) (**p < 0.01) but did not significantly differ between the groups. Error bars represent mean +/− SD.

Sperm incubated with glucose at 30 and 50 mM retained greater sperm vitality after 6 days, at 43.4% (*p* < 0.001) and 51.4% (*p* < 0.001) respectively, compared with SPM (Sperm Preparation Media) samples at 10% vitality (Fig. 2A).

Sperm vitality was measured using the eosin-nigrosin stain after treatment with glucose (30 and 50 mM), MG (50 µM) and GO (50 µM) for 6 days. Sperm vitality decreased in all treatment groups at the end of the incubation period but the presence of glucose maintained vitality at a significantly higher level than Sperm Preparation Media, SPM (***p < 0.001). The presence of MG and GO also showed significantly higher vitality than SPM (*p < 0.05) (Fig. 2B). Total progressive sperm motility significantly decreased over time for all treatment groups (**p < 0.01) (Fig. 2C). However, there was no significant difference between sperm treated with glycating compounds and SPM, nor between mannitol-treated sperm and glucose-treated sperm.

#### Measurement of AGE levels

**Figure 3:**
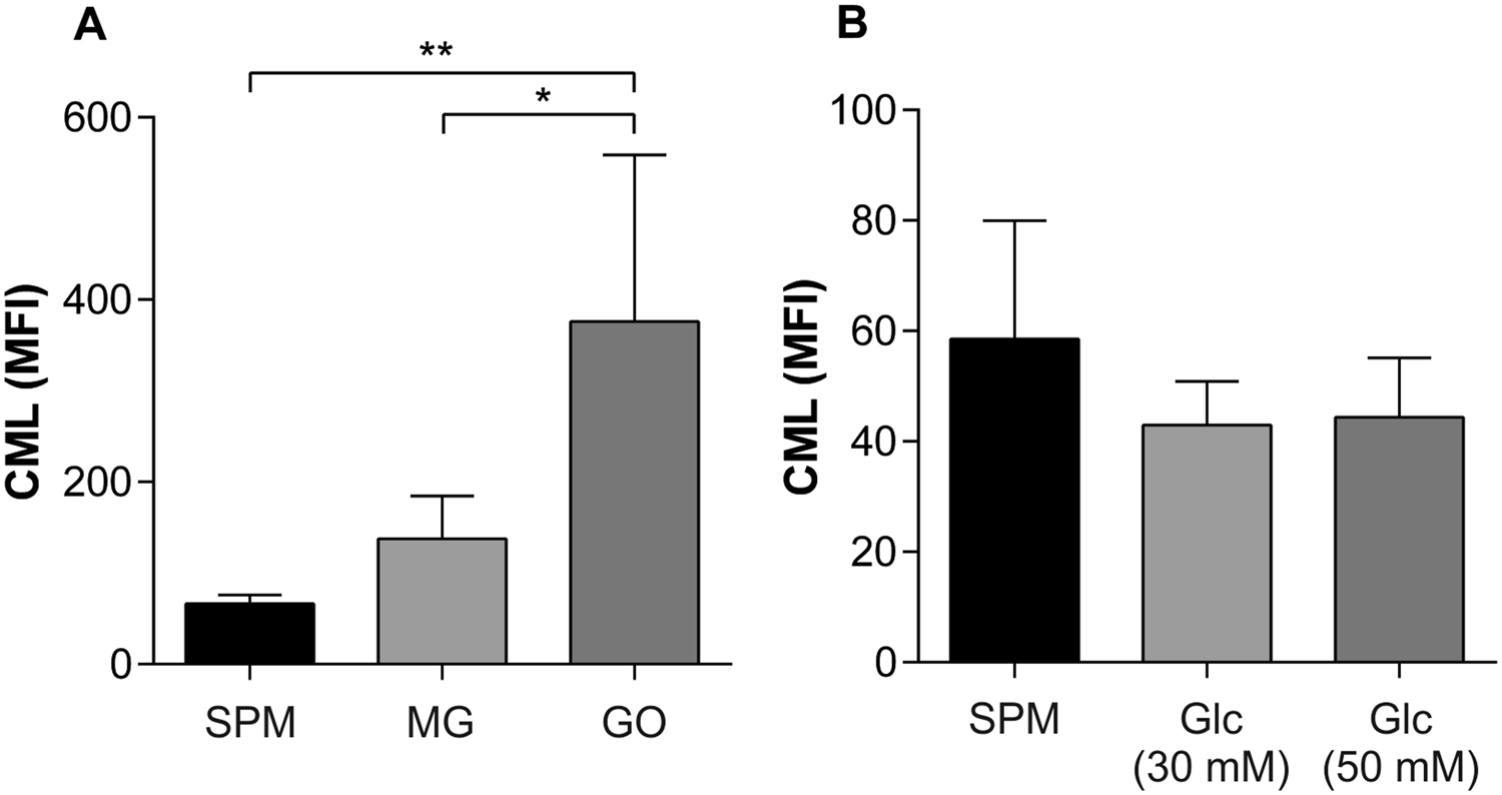
Carboxymethyl lysine formation in response to glycating agents. (**A**) Incubation of sperm with GO (50 µM) (n = 5) for 6 days resulted in a significant increase in the level (mean MFI +/− SD) of CML in comparison to SPM (n = 5) (*p* < 0.01) and MG (n = 5) (*p* < 0.05). (**B**) Incubation of sperm with 30 mM glucose (Glc) (n = 3) or 50 mM glucose (n = 3) had no effect on CML levels in comparison to SPM (n = 3). Error bars represent mean +/− SD.

Incubation of sperm with glucose and AGE intermediates GO and MG was carried out to examine the role of glycation on sperm function and whether AGE were formed in their presence. The level of CML, a prevalent AGE that has been previously detected on sperm cells^25^, was measured and found to be significantly elevated in response to GO (Fig. 3A). Sperm incubated with MG demonstrated no significant difference in the levels of CML compared to sperm treated with SPM or glucose. Glucose (30 mM and 50 mM) did not cause any change in CML compared to SPM. (Fig. 3B).

#### Immunolocalisation of AGE and CML

**Figure 4:**
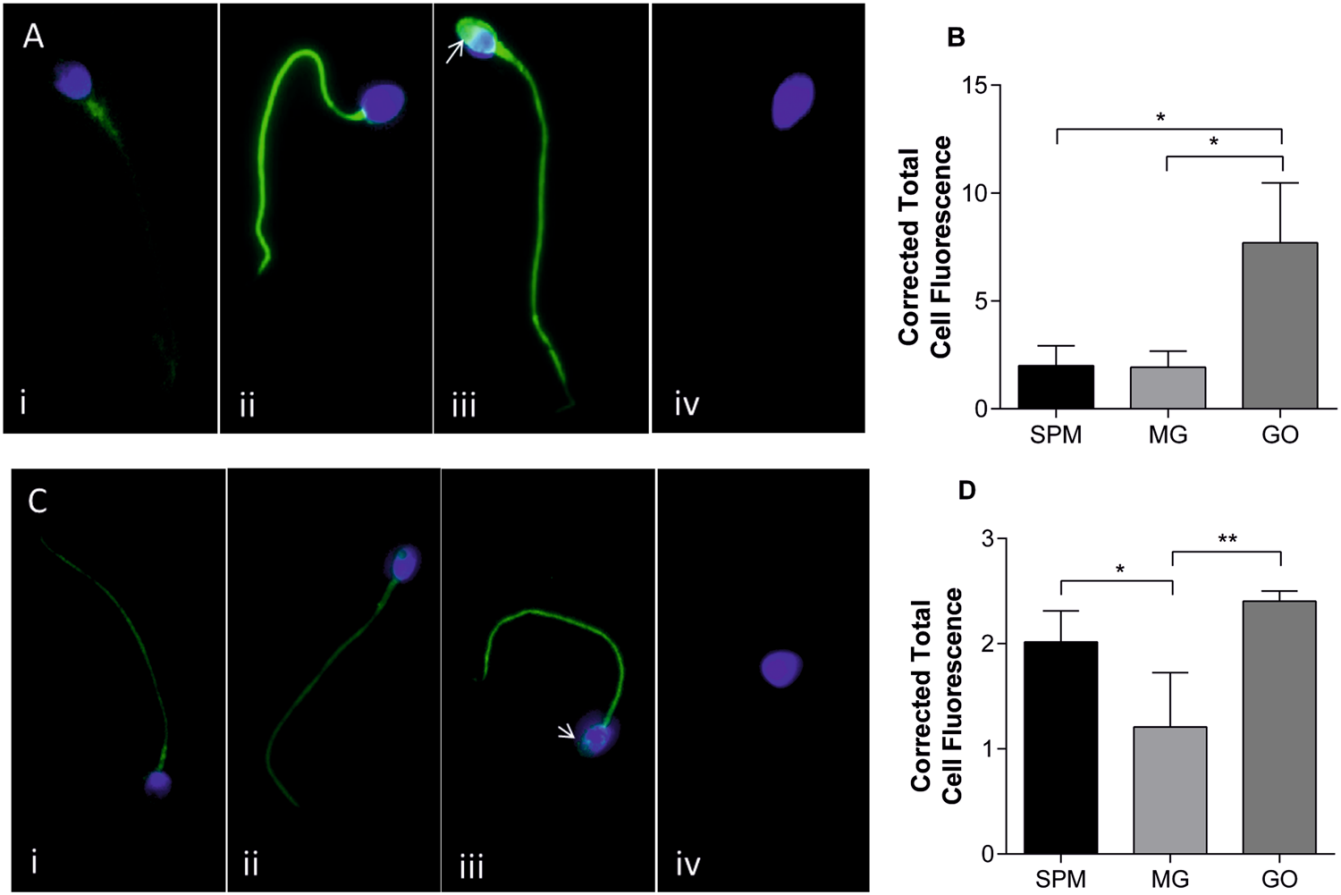
Immunolocalisation of CML and AGE on sperm. (**A**) Immunocytochemical staining of CML on SPM (i), MG (ii) and GO (iii) sperm revealed strong positive staining (green) on the tail of MG and GO sperm, and uniquely on the head region of sperm incubated with GO (arrow) (n = 3). Negative control showed no CML staining (iv). (**B**) Mean (+/− SD) corrected total cell fluorescence (CTCF) was calculated from 10 cells of different experimental replicates and showed CML to be significantly higher in the head region of GO sperm (*p* < 0.05). (**C** and **D**) General AGE staining (green) indicated the presence of AGEs on all treatment groups, with both GO (iii) and SPM (i) showing significantly higher head fluorescence than MG (ii) (arrow) (*p* < 0.01, *p* < 0.05, respectively) (n = 3). Magnification was at 100× oil immersion. Nuclear staining is shown in blue (DAPI).

To infer which sperm functions may be affected by glycation, the location and distribution of AGE on glycated sperm was determined by immunocytochemistry. CML-positive fluorescence could be seen strongly along the tail and midpiece regions of sperm treated with MG (Fig. 4Aii) and GO (Fig. 4Aiii), but less so with SPM (Fig. 4Ai). The Corrected Total Cell Fluorescence (CTCF) showed that in the head region, GO-treated sperm had significantly (*p < 0.05) higher CML fluorescence than SPM and MG (Fig. 4B). This fluorescence appeared strongly at the anterior part of the sperm head, encompassing the acrosomal region Fig. 4Aiii, arrow) (n = 3). Immunolocalisation of general AGE on sperm after glycation with GO, MG revealed some staining all treatment groups (Fig. 4C), with both GO and SPM showing significantly higher head fluorescence than MG after CTCF (Fig. 4D**)**.

#### Glycating compounds and oxidative stress

The present study has established AGE formation on sperm upon treatment with glycating agents *in vitro*. The effect of this observed AGE formation on ROS production and subsequent oxidative damage to sperm was then investigated.

**Figure 5:**
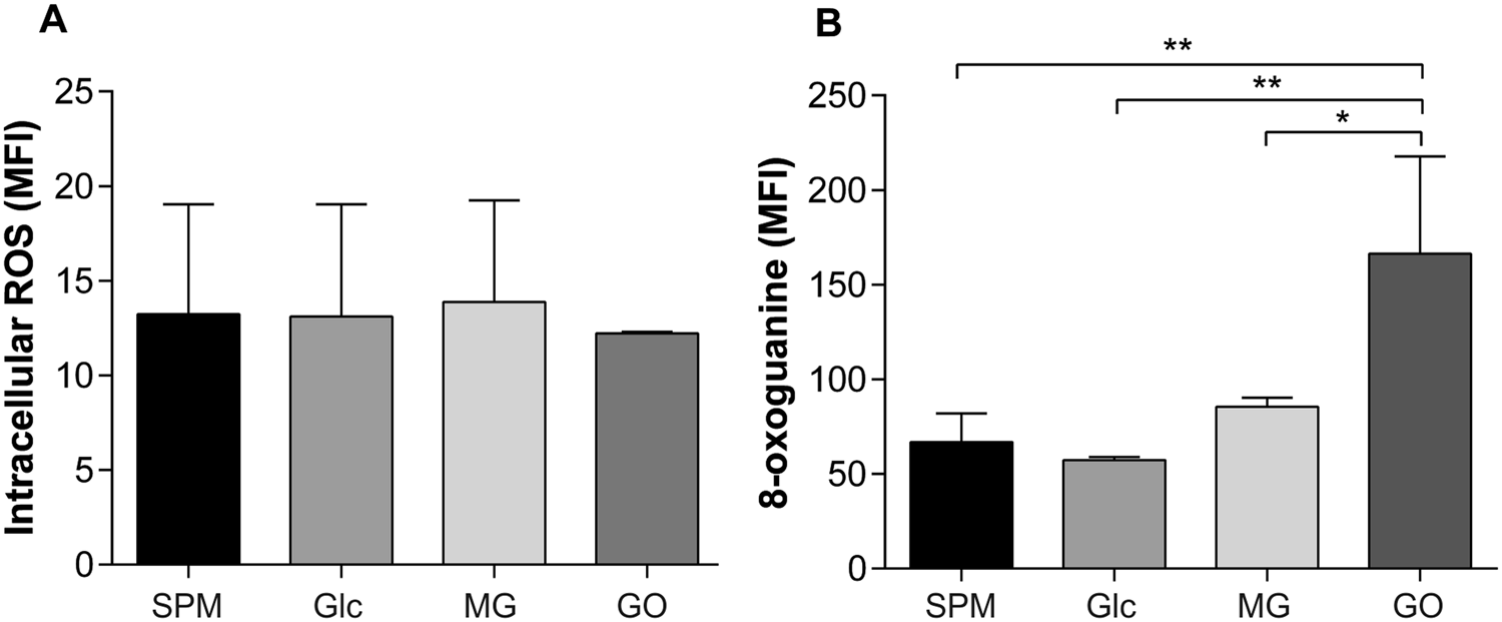
Intracellular ROS production and oxidative DNA damage. The effects of sugars and intermediate compounds on intracellular ROS levels in sperm. (**A**) Relative ROS levels in sperm treated with SPM (n = 3), glucose (Glc, 30 mM) (n = 3), MG (50 μM) (n = 3) or GO (50 μM) (n = 3) for 6 days. ROS is shown as mean fluorescence intensity (MFI) +/− SD of flow cytometry detection. One-way ANOVA analysis revealed no difference in ROS levels between any of the treatment groups. (**B**) 8-oxoguanine levels were measured in sperm treated with SPM (n = 3), glucose (Glc, 30 mM) (n = 3), MG (50 μM) (n = 3) and GO (50 μM) (n = 3). 8-oxoguanine levels are shown as MFI of flow cytometry detection. GO treatment caused significantly more 8-oxoguanine generation in comparison to MG (*p < 0.05), glucose (*p < 0.01) and SPM (*p < 0.01). Error bars represent mean +/− SD.

Intracellular ROS were measured in sperm after the long-term (6-day) glycation incubation period. Sperm were incubated with 30 mM glucose only, as previous treatments with both 30 mM and 50 mM showed no significant difference in AGE formation. The results showed no significant differences in ROS levels between sperm incubated with glucose or the intermediate glycating agents, GO and MG (Fig. 5A). Although differences in ROS levels were not detected, when an end-product of oxidative DNA damage, 8-oxoguanine, was measured, a significantly higher level was found in sperm incubated with GO in comparison to SPM (**p < 0.01) glucose (**p < 0.01) and MG (*p < 0.05) (Fig. 5B).

### Short term *in vitro* glycation

**Figure 6:**
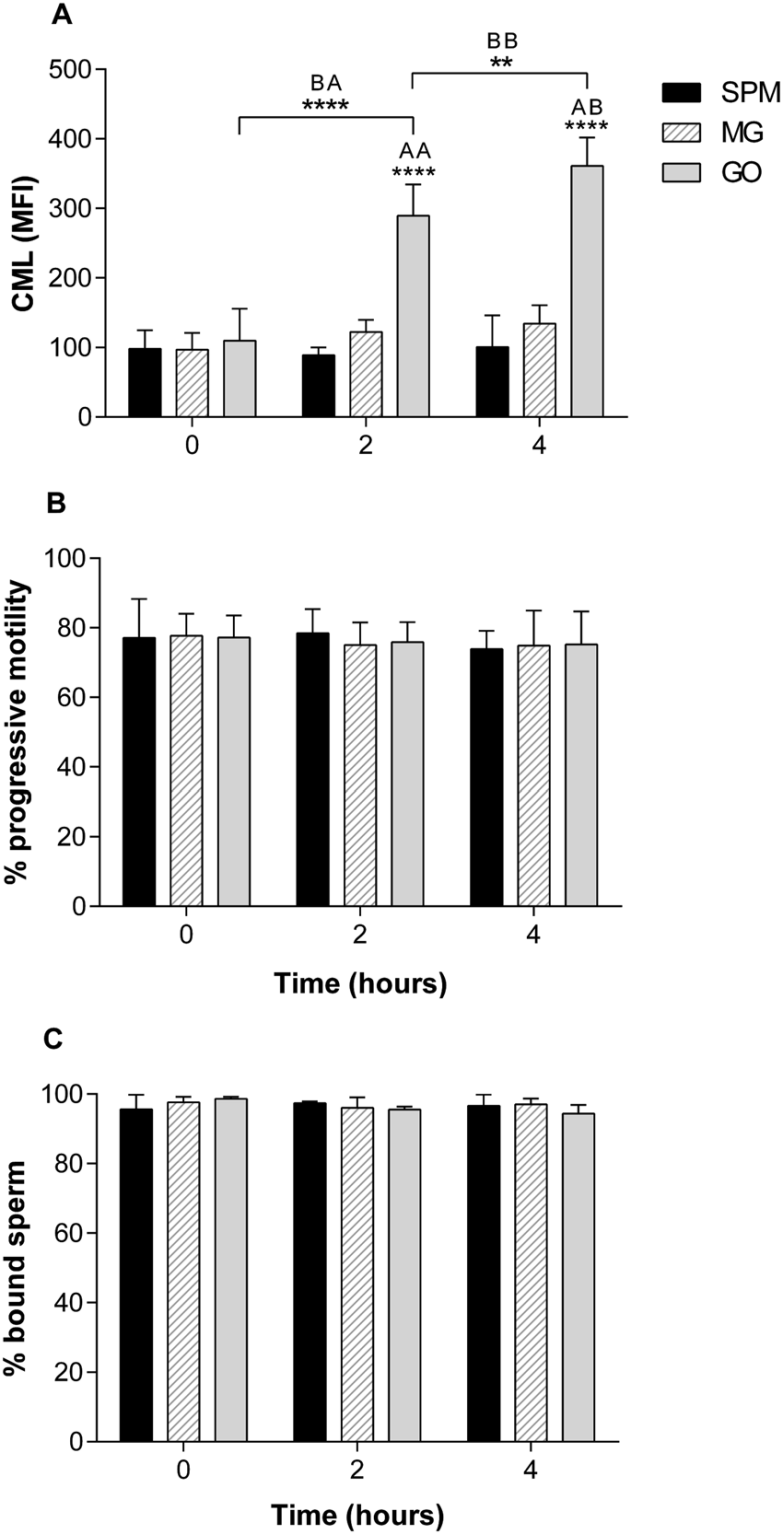
Short term glycation of sperm by GO. (**A**) CML levels (MFI +/− SD) in sperm treated with SPM, MG and GO (n = 3). Sperm incubated with GO showed an increase in CML at 2 hours (BA) and 4 hours (BB), both significantly higher than MG and GO at these time points (AA and AB). ****p < 0.0001, **p < 0.01. Key applies to (**A**), (**B**) and (**C**). (**B**) Sperm progressive motility (mean +/− SD) during glycation with SPM, MG and GO (n = 3). Two-way ANOVA showed no significant difference in progressive motility over 4 hours and between any treatment group. (**C**) HA binding of sperm incubated with MG and GO (n = 3). No difference was seen in hyaluronan binding capacity at 2 or 4 hours. Sperm maintained a high level of binding throughout. Error bars represent mean +/− SD.

As AGE intermediates react rapidly and form more readily than reducing sugars such as glucose, CML formation was measured over a four hour time period, rather than 6 days as in previous experiments. Exposure to GO caused rapid generation of CML after only two hours in culture, which increased further at four hours (Fig. 6A). This was significantly greater than CML levels from SPM- and MG-treated cells (*p* < 0.001). Importantly, the rapid increase in CML levels did not correlate with a change in sperm progressive motility (Fig. 6B).

As sperm interact with a number of ligands during fertilization, the effect of the observed CML formation on Spam1, an important membrane protein in sperm-oocyte interaction, were investigated^33,34^. Sperm hyaluronidase activity showed no significant change following 4 hours of *in vitro* glycation with MG or GO. (Fig. 6C).

## Discussion

Glycation is the spontaneous non-enzymatic reaction of a carbonyl group of a reducing sugar with a protein amine group, which through multiple successive reactions forms covalent irreversible AGEs.

AGEs and the reactive intermediates such as MG can covalently adduct to proteins, which may induce structural and functional changes that could alter molecular conformation and enzymatic activity. AGEs can also increase inflammation and oxidative stress by forming ROS^35^ through interactions with the RAGE. MG also disrupts the electron transport chain in mitochondria^36^.

As AGE have been located on sperm cells and in the male reproductive tract^25^, this study sought to use an *in vitro* experimental model to investigate possible effects of these compounds on sperm function. The reducing sugar glucose was used as a physiologically relevant inducer of AGE formation, and the reactive AGE intermediates MG and GO, were used to illustrate the rapid glycating effect of these compounds. The main findings of this study were that CML, a common and potent AGE was rapidly formed on sperm when incubated with GO. Glycated sperm experienced higher levels of 8-oxoguanine formation, indicating a role for AGE-mediated DNA damage.

Over a 6-day period in culture, CML levels did not increase in sperm incubated with glucose. This low reactivity of glucose is rational given its role as the most abundant sugar within biological systems; high glucose reactivity would cause inevitable damage to biomolecules. This result was unsurprising as many studies investigating the chemical processes of glucose-mediated glycation have shown that this can take from 1 to 4 weeks, often as long as the turnover time of the proteins *in vivo*^37–39^. Furthermore, a recent study demonstrated that sperm parameters, such as motility and viability were unaffected by exposure to high levels of glucose *in vitro*^40^.

As sperm are haploid, non-dividing cells, they can only be maintained in culture for limited periods (<1 week), and as glycation is directly dependent on glucose concentration and time^39^, glucose was used at concentrations higher than normal physiological levels. Under healthy conditions, glucose levels are between 4–6 mM in blood and 1 mM in seminal plasma, while hyperglycaemic type 2 diabetics experience blood glucose levels of 11 mM up to and sometimes above 30 mM. In this glycation assay, neither 30 mM nor 50 mM glucose increased CML levels after 6 days. It is also possible that the lack of CML formation in response to glucose was due to sperm metabolising the glucose in solution, causing a decrease in concentration to levels insufficient for glycation. In some fertility clinics, sperm are maintained in culture media such as SPM for a number of hours. This medium is optimised for sperm function and contains glucose at approximate levels of less than 1 mM (http://www.origio.com), which the results of this study suggest would be insufficient for glycation-mediated damage to occur to sperm^41^.

The AGE intermediates GO and MG are reactive carbonyl compounds (RCCs). They are formed during the Maillard reaction but also from the oxidation of AGEs^7^. RCCs rapidly form Schiff bases with amino acids, hence their greater reactivity than glucose, and eventually form imidazolone adducts with molecules, which can cause cytotoxicity to different cell types^42^. This *in vitro* study showed that exposing sperm to GO for as little as 2 hours caused a significant increase in CML levels compared with SPM and MG.

Although the concentrations of MG and GO used here were higher than normal physiological levels which are approximated to be 100–120 nM in blood plasma^7^, high levels were used to illustrate the potential effects of these compounds at a measurable level, and in line with, if not lower than, those used in other *in vitro* cell assays^43^.

Immunocytochemical staining of glycated sperm was carried out in order to demonstrate the distribution of general AGEs and the more specific AGE, CML on the sperm cells after exposure to glycating compounds *in vitro*. The staining of CML along the entire length of the cell, including head, midpiece and tail is in accordance with the staining pattern found by Mallidis *et al*.^25^. They also described high immunoreactivity to CML on the head acrosomal cap region in diabetic men compared to non-diabetics where immunoreactivity was low. This is similar to the higher level of CML staining found here on sperm from healthy men glycated with GO compared with SPM when quantified using corrected total cell fluorescence (Fig. 4A,B). Although caution must be taken when extrapolating *in vitro* data with *in vivo* studies, the use of this *in vitro* assay may be a good model for mimicking the diabetic environment.

To explore the immunocytochemical staining of general AGE compounds on sperm, an anti-AGE antibody, targeting general AGE compounds was used. This revealed no significant difference between GO treated and control (SPM) sperm (Fig. 4C,D), in contrast to the CML-specific staining (Fig. 4A,B), suggesting that the changes we see are more specific to CML than all AGEs in general. It is interesting to note that in MG treated sperm there was actually a decrease in general AGE labelling compared to control in contrast to the CML data. Importantly, as AGEs are heterogeneous, the general anti-AGE antibody used can react with a wide number of glycated compounds and the degree of labelling might differ as a result of the affinity of the anti-AGE antibody. Thus, as CML is the physiological AGE associated with AGE-related pathologies it was important that we focused more on the effects of this AGE compound.

GO and MG have been shown to cause rapid generation of CML on proteins such as BSA and lysozyme *in vitro*^37,42^. Therefore, the CML production seen in the first four hours on sperm is possibly occurring on structurally available proteins such as receptors on the sperm membrane, while later CML generation could be occurring as a result of adduct formation on intracellular proteins and DNA. Although AGE formation was originally thought to only affect stable extracellular proteins, such as collagen, it is now clear that glycation adducts can quickly form on intracellular proteins such as actin^44^ and on DNA^45^.

Cell-based *in vitro* glycation assays have previously shown that cell receptors such as the platelet derived growth factor receptor are vulnerable to glycation^39^. Critically, modification of these receptors by AGE intermediates can alter their function, as was shown through inhibited epidermal growth factor receptor signalling^46^. Sperm participate in a number of receptor-ligand interactions to which glycation could be detrimental, such as for the hyaluronidase activity of *Spam1*, which is responsible for degradation of HA oligosaccharide chains found in the cumulus layer of granulosa cells surrounding the oocyte^34^. *Spam1* is located on the acrosomal membrane where a high level of CML immunoreactivity was observed in this study, and as this protein contains a number of lysine and arginine residues, this enzyme could be affected by glycation. Therefore, the function of sperm hyaluronidase binding was investigated in response to glycation.

Sperm binding to HA was not affected following short-term glycation. As the hyaluronan binding assay requires motile sperm in order to distinguish between sperm that are bound (hyaluronidase active) and unbound (hyaluronidase inactive), the experiment could not be carried out at 6 days when sperm motility is compromised^47^.

The oxidized guanine nucleotide, 8-oxoguanine, is the most common DNA lesion that occurs as a result of oxidative damage by ROS. The lesion proceeds to destabilise the DNA structure through altered oxoGC pairing. In the present study, 8-oxoguanine was found to be increased in sperm with elevated CML levels, suggesting that oxidative DNA damage occurs alongside AGE formation. AGE-related ROS generation can happen in a number of ways. Firstly, AGE intermediates can cause ROS generation during the glycation process, as well as independently of AGE formation^44^. Therefore, the process of CML formation seen here is likely to be triggering ROS formation, such as has been demonstrated in endothelial cells, and which is inhibited in the presence of radical-scavenging enzymes^45^. Another major source of ROS arises through AGE binding to their receptor, RAGE, on the cell membrane. AGE-RAGE binding is important in AGE pathology in various tissues. Furthermore, RAGE has also been located on the sperm membrane and its expression positively correlates with DNA damage^25,26^. Importantly, both of these parameters are elevated in the sperm of diabetic men^31^. Although mature sperm are transcriptionally quiescent, which may suggest a limited capacity for NFκB cell signalling, immature sperm in the seminiferous tubules still have active gene expression and would be vulnerable to ROS generation through this pathway.

Despite glycation being associated with oxidative DNA damage, ROS levels were not different between sperm incubated with the different glycating compounds. This may be due to the extensive experimental time period, in which case ROS generation would be at a maximum across all samples as cell death began to occur. This effect would have been increased as a result of the absence of seminal plasma, the source of antioxidants and radical scavenging enzymes important for sperm protection^48,49^. Additionally, as ROS are unstable, a more sensitive assay may be required to detect low levels of ROS.

Sperm are particularly susceptible to DNA damage, which occurs primarily due to oxidative stress in these cells^49^ as they lack the repair mechanisms present in somatic cells. Furthermore, the glyoxalase pathway, which detoxifies AGE intermediates through the action of Glyoxalase 1^50^ has not been identified in sperm. The implications of such damage for sperm *in vivo* are reduced cell function, ultimately leading to poorer fertilisation capacity. 8-oxoguanine levels have also been linked to lower pregnancy rates independent of semen quality^51,52^. This study found no significant change in the motility of sperm treated with GO where oxidative DNA damage was highest. However, oxidative DNA damage can occur in normal and abnormal sperm, and as ART treatment does not take into account the analysis of DNA damage routinely, this may have consequences for embryonic development and offspring health^53,54^.

## Conclusion

The present study shows that AGE compounds are formed on human sperm upon exposure to the reactive GO *in vitro*, leading to a significant increase in the oxidative DNA adduct, 8-oxoguanine. Importantly, there was no significant impacts on sperm motility or sperm hyaluronan binding after exposure to the glucose or the reactive AGE intermediates.

In summary, this study demonstrates that glycation induces sperm DNA damage without evident differences in sperm function when tested for changes in standard semen parameters.

## Materials and Methods

### Human Biological Material

#### Human Biological Material

Human semen samples were obtained from seven healthy normospermic, non-diabetic, consenting donors (aged 19–35 years). All donors signed informed consent and samples were processed in accordance with faculty ethical approval at Manchester Metropolitan University. Semen samples were obtained via masturbation after 3–5 days of sexual abstinence for provision of quality samples, in accordance with recommendations by the World Health Organization (WHO)^55^. This minimised variation in sample quality and sperm DNA integrity that occurs with length of abstinence^56^.

Semen samples were allowed time for liquefaction to complete before further analysis and preparation. Density gradient centrifugation was used to isolate sperm from seminal plasma. Briefly, 1 ml of semen was layered on top of a gradient of 55% and 80% SupraSperm^TM^ media (Origio, Denmark) and centrifuged at 300 *g* for 20 minutes. The supernatant was removed and discarded and the sperm pellet was washed twice in Sperm Preparation Media^TM^ (SPM, Origio, Denmark), by centrifugation at 300 *g* for 5 minutes. The cells were then resuspended in 1 ml SPM. All downstream experiments were carried out with washed, semen-free sperm.

### *In vitro* glycation assays

To establish the effect glycating agents have on sperm vitality and motility in an *in vitro* system, sperm were incubated with various glycating compounds over long-term (6 days) and a short-term incubation periods (0, 2 and 4 hours). 3 donors were used for the long-term experiments, and samples from another set of 3 donors were used for the short-term experiments. Long-term experiments were carried out to show the glycating effects of glucose, which has low reactivity relative to glucose-6-phosphate^57^ or other reducing sugars such as D-ribose^58^. A period of 6 days was chosen as glucose has previously shown no glycating effects on cultured cells over 2 days^58^. Mannitol - a non-reducing sugar - was included in initial experiments to control for osmotic effects of glucose^59^. The highly reactive AGE intermediates, MG and GO, were used as an accelerated model for the glycation of sperm. Cellular levels of these compounds are predicted to be approximately 1–5 µM and 0.1–1 µM, respectively^60^. However, physiological levels of MG have been reported to be in the nM^61^, µM^62^ and mM^63^ range. The concentrations implemented in this study were higher in order to simulate a disease environment and were in line with those used in other studies^39^.

For the long-term *in vitro* glycation experiments, motile sperm separated by density gradient centrifugation were pelleted and resuspended at a concentration of 20 × 10^6^/ml in solutions of SPM containing D-glucose (30 mM or 50 mM), Mannitol (30 mM). These concentrations were used to mimic diabetic hyperglycemia and to asses sperm response to massive doses of glucose^40^. Sperm were also exposed to methylglyoxal (MG) (50 μM), glyoxal (GO) (50 μM) or SPM alone. Sperm were incubated at 34 °C on a rotator for 6 days before analysis of CML formation, ROS levels and oxidative DNA damage. Incubations were carried out at a temperature of 34 °C in order to best represent the scrotal environment of normospermic men^64^. Sperm vitality was assessed after the long-term incubation experiments using the eosin-nigrosin stain as outlined above and motility was measured using the CASA^65^.

For the short-term *in vitro* glycation experiments, sperm at a concentration of 20 × 10^6^/ml were treated with solutions of SPM containing MG (50 μM) and GO (50 μM); cells were removed at 0, 2 and 4 hour time points for analysis of motility, CML formation and hyaluronan binding capacity.

#### Motility assessment

To measure sperm motility for both short-term and long-term glycation experiments,, 5 μl sperm from each sample was applied to a cell counting slide in duplicate (CellVision®, Mitrone) and analysed by a Computer Assisted Sperm Analyser (CASA – Sperminator®, Procreative). Automated CASA technology allowed standardization of measurements, as used elsewhere^65,66^. Three CASA measurements were recorded for each sample and 200 sperm were analyzed per read^67^. For the long-term glycation experiments - progressive sperm motility was assessed at three different time points (24, 48 and 72 hours) as a period of 3 days was chosen as the longest period that could be used without any major loss of sperm motility in the control sample^47^.

#### MG toxicity assay

The concentration of MG and GO to use in the glycation experiments was determined using a toxicity assay in which sperm (20 × 10^6^/ml) were incubated with 0, 50, 100, 250 and 500 μM MG in SPM for 48 hours at 34 °C. Due to the limited availability of donor samples, the toxicity assay was carried out on a single sample. Vitality was measured using the one-step eosin-nigrosin stain. The stain was prepared as described in WHO 5^th^ edition^55^ Briefly, eosin Y (0.67%) (Sigma, UK) and sodium chloride (NaCl) (0.9%) (Sigma, UK) were dissolved in water with gentle heating, before nigrosin (10%) (Sigma, UK) was added, and the solution was boiled, then allowed to cool and filtered. Equal volumes of eosin-nigrosin stain and resuspended sperm sample were mixed and smeared onto a microscope slide using the feathering technique. Duplicate smears were made for each concentration of MG using a fresh aliquot of sperm. Using a light microscope (100 × oil objective), 200 sperm were counted on each slide and the average number of live sperm was recorded. The percentage of live sperm were calculated as [# live cells/# total cell count] × 100.

The chosen working concentration of MG was 50 µM as the toxicity assay showed only 8% cell death over the course of the experiment, in comparison to 28% and 77% cell death at 100 µM and 500 µM MG, respectively.

#### Measurement of CML

The level of CML on sperm was measured by flow cytometry after both long-term and short-term *in vitro* glycation, as has been demonstrated elsewhere^68^.

Sperm were removed from glycating solutions after long-term (n = 3) and short-term periods (n = 3) and pelleted by centrifugation at 300 *g* to remove glycation media. Sperm cells were resuspended in paraformaldehyde (4%) (Sigma, UK) and incubated at room temperature on a tube rotator (Eppendorf, UK) for 20 minutes. Cells were pelleted again and permeabilized by resuspension in 0.1% PBS-tween for 15 minutes. Sperm were then blocked in 10% normal goat serum (Vector laboratories, UK) in 1xPBS for 1 hour at room temperature. Cells were incubated with a mouse anti-CML primary antibody (1:50) (Abcam, UK, ab125145) for one hour at room temperature, followed by a goat anti-mouse Alexa Fluor 488 secondary antibody (1:2000) (Life Technologies, UK) for 45 minutes at room temperature in the dark. Cells were then washed once and resuspended in 1xPBS before acquisition using a BD FACSCalibur™ (BD Biosciences, UK) and analysed with BD CellQuest™ Pro software (BD Biosciences, UK). Cells were gated and debris was excluded in the forward scatter (FSC) and side scatter (SSC) plot. Fluorescein isothiocyanate (FITC) fluorescence was detected in the FL-1 channel (488 nm). Each test sample was measured in triplicate and 10,000 events were counted each time. A FITC-negative cell population was gated using an unstained control sample in the FL-1 channel. Relative CML levels were recorded as the mean fluorescence intensity (MFI) of cells.

#### Measurement of intracellular reactive oxygen species

Spontaneous glycation of biomolecules and later stages of the Maillard reaction are associated with the generation of ROS and oxidative stress. Flow cytometric methods have been established for numerous evaluation parameters in sperm^69^, including for ROS measurement. Intracellular ROS in sperm was measured after long-term glycation incubation (n = 3) with either glucose, MG or GO using the CM-H2DCFDA General Oxidative Stress Indicator (Invitrogen, UK). CM-H2DCFDA diffuses into cells where it is oxidized to yield a fluorescent adduct, measured in the fluorescein spectrum^70,71^. The assay was carried out as instructed by the manufacturer. Briefly, treated sperm were washed in pre-warmed PBS and resuspended in PBS containing the ROS probe. The cells were incubated at 34 °C for 30 minutes. The dye was then removed by centrifugation and the cells resuspended in PBS and FITC-positive cell fluorescence was detected by flow cytometry under the same conditions as for CML measurement, in the FL-1 channel. A negative control of sperm unexposed to the dye was made and a positive control of sperm exposed to H_2_O_2_ for 15 minutes prior to the assay to induce oxidative damage. These controls were used to gate the fluorescence-negative and -positive cells prior to ROS measurement. 10,000 cells were counted in triplicate for each experimental sample (n = 3) and relative ROS levels were expressed as MFI values.

#### Measurement of 8-oxoguanine

The level of oxidative DNA damage in spermatozoa incubated with glycating agents over 6 days (n = 3) was determined using the fluorometric OxyDNA Assay Kit (Calbiochem®, EMD Millipore, US). The assay is based on a FITC-conjugate that binds to the 8-oxoguanine moiety of oxidized DNA, as performed on sperm cells elsewhere^69^. Sperm cells were centrifuged at 300 *g* for 7 minutes to pellet the cells and remove media. Cells were fixed in 4% PFA and permeabilised with 70% ethanol. Cells were washed with Wash Solution (1×) provided with the kit and resuspended in 100 μl FITC-conjugate (1×), before incubation in the dark for 1 hour at room temperature. Cells were washed again in Wash Solution (1×), resuspended in 1xPBS and analyzed by flow cytometry as for CML. Three flow cytometry measurements were made for each experimental sample (n = 3).

#### Immunocytochemical staining of sperm for AGE and CML

To identify the localization of AGE formation on sperm, samples from the long-term incubation experiment (n = 3) were stained for AGEs using a general anti-AGE antibody^72^ (Abcam, UK, ab23722) and specifically for CML using an anti-CML antibody^73^ (Abcam, UK, ab125145). Sperm that had been incubated with SPM, MG and GO were stained for AGEs and CML. 10 μl of sperm was placed on one end of a glass microscope slide and smeared across the slide using the feathering technique and allowed to air dry. Slides were then fixed by submersion in ice-cold methanol (100%) for 15 minutes. Once dry, a water-resistant pen (Life technologies, UK) was used to seal the area around the cells. Slides were rehydrated in PBS-tween (PBS-T) for 3 minutes before blocking in 10% normal goat serum in PBS-T (0.05%) for 1 hour at room temperature. Slides were washed in 3× fresh changes of PBS-T for 1 minute each. Primary antibodies were diluted in PBS-T (1:200), added to the slides and incubated in a humidified chamber overnight at 4 °C. Negative controls were included in which the primary antibody was omitted. The following day, slides were washed in 3× changes of PBS-T and FITC-conjugated goat anti-mouse (1:2000) or goat anti- rabbit (1:2000) secondary antibodies were added to the slides staining for CML and AGEs, respectively. Slides were incubated with secondary antibodies for 1 hour at room temperature in a darkened humidified chamber. Slides were washed a final 3 times in PBS-T before excess reagent was drained off and sperm nuclei were stained for using Vectashield Mounting Medium with DAPI (Vectashield, UK) and finally coverslipped. Fluorescent images were taken using an Axio Imager Z1 (Zeiss, HBO 100 mercury lamp) with AxioVision 4 software. Sperm head fluorescence was quantified using ImageJ software and the Corrected Total Cell Fluorescence (CCF)^74^ was obtained using the equation CCF = initial cell density − (cell area × background intensity).

#### Sperm hyaluronan binding assay

Functional activity of plasma membrane hyaluronidase in glycated and non- glycated sperm was measured using Hyaluronan Binding Assay (HBA) slides^75^ (Origio, Denmark). At 0, 2 and 4 hours of incubation with MG, GO or SPM, 8 μl of sperm suspension was removed and placed onto a hyaluronan-coated slide and a coverslip was applied. After a 10-minute incubation period in which sperm were allowed to bind to hyaluronan, the number of bound motile sperm and the number of unbound motile sperm on a 10 × 10 square grid were counted at 40 × magnification. Immotile sperm were not counted. Bound sperm are differentiated from unbound sperm by their beating tails with heads that make no progressive movement. Sperm motility was also assessed at these stages using the CASA recording grades A (fast progressive motility), B (slow progressive motility), C (non-progressive motility) and D (immotile).

#### Statistical analysis

Statistical analysis was carried out using GraphPad Prism 6 Software (Version 6.01). Descriptive statistics were analyzed for each variable, observing means and standard deviation (SD). Ordinary one-way ANOVA and Tukey’s multiple comparisons tests were used to identify significant differences across different treatment groups in both short-term and long-term glycation experiments. A repeated measures ANOVA and Tukey’s multiple comparisons tests were used to test statistical significance across treatment groups and over multiple time points. Statistical significance was established when the above tests returned a *p*-value < 0.05.
